# Efficacy and Acceptability of Interventions for Attenuated Positive Psychotic Symptoms in Individuals at Clinical High Risk of Psychosis: A Network Meta-Analysis

**DOI:** 10.3389/fpsyt.2018.00187

**Published:** 2018-06-12

**Authors:** Cathy Davies, Joaquim Radua, Andrea Cipriani, Daniel Stahl, Umberto Provenzani, Philip McGuire, Paolo Fusar-Poli

**Affiliations:** ^1^Early Psychosis: Interventions and Clinical-Detection Lab, Department of Psychosis Studies, Institute of Psychiatry, Psychology and Neuroscience, King's College London, London, United Kingdom; ^2^FIDMAG Germanes Hospitalàries, CIBERSAM, Barcelona, Spain; ^3^Department of Clinical Neuroscience, Centre for Psychiatry Research, Karolinska Institutet, Stockholm, Sweden; ^4^Department of Psychiatry, University of Oxford, Oxford, United Kingdom; ^5^Oxford Health NHS Foundation Trust, Oxford, United Kingdom; ^6^Department of Biostatistics, Institute of Psychiatry, Psychology and Neuroscience, King's College London, London, United Kingdom; ^7^Department of Brain and Behavioral Sciences, University of Pavia, Pavia, Italy; ^8^Department of Psychosis Studies, Institute of Psychiatry, Psychology and Neuroscience, King's College London, London, United Kingdom; ^9^National Institute for Health Research Maudsley Biomedical Research Centre, London, United Kingdom; ^10^OASIS Service, South London and Maudsley NHS Foundation Trust, London, United Kingdom

**Keywords:** psychosis, risk, interventions, symptoms, network meta-analysis, treatments

## Abstract

**Background:** Attenuated positive psychotic symptoms represent the defining features of the clinical high-risk for psychosis (CHR-P) criteria. The effectiveness of each available treatment for reducing attenuated positive psychotic symptoms remains undetermined. This network meta-analysis (NMA) investigates the consistency and magnitude of the effects of treatments on attenuated positive psychotic symptoms in CHR-P individuals, weighting the findings for acceptability.

**Methods:** Web of Science (MEDLINE), PsycInfo, CENTRAL and unpublished/gray literature were searched up to July 18, 2017. Randomized controlled trials in CHR-P individuals, comparing at least two interventions and reporting on attenuated positive psychotic symptoms at follow-up were included, following PRISMA guidelines. The primary outcome (efficacy) was level of attenuated positive psychotic symptoms at 6 and 12 months; effect sizes reported as standardized mean difference (SMD) and 95% CIs in mean follow-up scores between two compared interventions. The secondary outcome was treatment acceptability [reported as odds ratio (OR)]. NMAs were conducted for both primary and secondary outcomes. Treatments were cluster-ranked by surface under the cumulative ranking curve values for efficacy and acceptability. Assessments of biases, assumptions, sensitivity analyses and complementary pairwise meta-analyses for the primary outcome were also conducted.

**Results:** Overall, 1,707 patients from 14 studies (57% male, mean age = 20) were included, representing the largest evidence synthesis of the effect of preventive treatments on attenuated positive psychotic symptoms to date. In the NMA for efficacy, ziprasidone + Needs-Based Intervention (NBI) was found to be superior to NBI (SMD = −1.10, 95% CI −2.04 to −0.15), Cognitive Behavioral Therapy-French and Morrison protocol (CBT-F) + NBI (SMD = −1.03, 95% CI −2.05 to −0.01), and risperidone + CBT-F + NBI (SMD = −1.18, 95% CI −2.29 to −0.07) at 6 months. However, these findings did not survive sensitivity analyses. For acceptability, aripiprazole + NBI was significantly more acceptable than olanzapine + NBI (*OR* = 3.73; 95% CI 1.01 to 13.81) at 12 months only. No further significant NMA effects were observed at 6 or 12 months. The results were not affected by inconsistency or evident small-study effects, but only two studies had an overall low risk of bias.

**Conclusion:** On the basis of the current literature, there is no robust evidence to favor any specific intervention for improving attenuated positive psychotic symptoms in CHR-P individuals.

## Introduction

Indicated prevention in people at Clinical High Risk for Psychosis (hereafter CHR-P) (1) represents one of the first attempts to alter the course of the most severe psychiatric disorder and thereby improve the lives of many young people (2, 3). Recent meta-analytical evidence has suggested that it is potentially the only effective way to reduce the duration of untreated psychosis, which is a key factor determining outcomes (4). CHR-P individuals accumulate several risk factors for psychosis (5), leading to subtle symptoms (6) and functional impairments (7) that trigger help-seeking behaviors (8). CHR-P individuals have around 20% risk [eTable 4 from Fusar-Poli et al. (9)] of developing psychosis [but not any other non-psychotic disorder (10, 11)] at 2 years. After two decades of CHR-P research, the paradigm is at standstill (12). The principal limitations of knowledge are: (i) poor penetrance of detection strategies for identifying at-risk individuals (13, 14), (ii) the prognostic accuracy of CHR-P tools in clinical use (15) being substantially dependent on idiosyncratic sampling and recruitment strategies (16–20), and (iii) an unclear effect of preventive treatments. Our research group has previously addressed the first two limitations, and only more recently have we completed a meta-analysis that has investigated the consistency and magnitude of the effects of treatments to prevent psychosis in CHR-P individuals. We used a network meta-analytic approach, which allows head-to-head comparisons to be performed across different preventive treatments, and which is the recommended evidence synthesis method for informing treatment guidelines (21). The key result of our analysis was that there is no evidence to favor any specific preventive treatment for CHR-P individuals over any others (22). This finding is not completely surprising, given that all of the most recent trials in this area were negative (23–31). Therefore, currently, there is no convincing evidence that indicated interventions implemented in CHR-P individuals can effectively prevent the onset of psychosis. The impact of available preventive interventions on the underlying neurobiology that characterizes the CHR-P state and the onset of psychosis is similarly unclear (3). We have fully discussed these findings and the limitations of our analysis in our previous report (22). Here, we complement our previous meta-analysis by focusing on outcomes other than the onset of new psychotic disorders. It is indeed apparent that CHR-P individuals may present with problems other than the development of psychosis at follow-up, such as the persistence of subthreshold psychotic symptoms (32). In particular, attenuated positive psychotic symptoms represent the defining features of the core CHR-P criteria. Meta-analytical evidence indicates that around 85% (95% CI 79% to 90%) of CHR-P individuals meet the intake criteria because of attenuated positive psychotic symptoms [see eFigure 1 in Fusar-Poli et al. (9)]. The severity and frequency of attenuated positive psychotic symptoms are carefully measured by experienced clinicians using specific semi-structured [and not self-administered (33)] CHR-P instruments (34). Investigating the effect of treatments on attenuated positive psychotic symptoms may also be associated with empirical research benefits. For example, it has been suggested that using continuous outcomes -such as attenuated positive psychotic symptoms- rather than the binary transition to psychosis may overcome the problems of arbitrary thresholds defining a categorical onset of psychosis (35). Investigating the impact of interventions on attenuated positive psychotic symptoms is also relevant for informing clinical guidelines. For example, the National Institute for Health and Care Excellence (NICE) guidelines recommend cognitive behavioral therapies (CBT) for presenting symptoms (36), but there is no clear evidence which can reliably support this recommendation. The other relevant outcome for CHR-P individuals is the acceptability of treatments. Given the relatively high proportion of false positives with respect to transition to psychosis, it is essential that treatments have a benign side effect profile, are well tolerated and acceptable to this patient group.

To address these gaps in knowledge, we present here a network meta-analysis investigating the consistency and magnitude of the effects of preventive treatments for reducing attenuated positive psychotic symptoms in CHR-P individuals, weighting the findings for acceptability. We focus on randomized controlled trials (RCTs) to avoid the selection biases associated with observational studies. Our primary aim was to test whether any specific treatments are any more or less effective (compared to any others) in improving attenuated positive psychotic symptoms in CHR-P individuals, and to provide an evidence-based ranking of treatments on the basis of efficacy and acceptability. We intended that this work would contribute to the rigorous evidence-based assessment of the strengths and limitations of the CHR-P paradigm (12). Our overarching vision is that by understanding the limitations of current knowledge—which is an essential prerequisite to finding ways of overcoming them—the CHR-P field can advance with the development of refined approaches that may ultimately achieve an effective prevention of psychosis.

## Methods

### Included interventions

In a first step we listed the preventive interventions of interest. The current study included all RCTs involving non-pharmacological and/or pharmacological interventions administered to CHR-P individuals. We focused on the following types of treatments: CBT (different protocols), integrated psychological therapies, psychoeducational interventions, supportive counseling, family therapy, needs-based interventions (NBI), antipsychotic molecules (aripiprazole, ziprasidone, risperidone, olanzapine) and any novel/experimental therapeutics (D-serine and omega-3 fatty acids). Although these interventions had been defined *a priori*, we also allowed the inclusion of additional treatments that were emerging from the most recent literature search. In a second step, we carefully reviewed the available systematic reviews and meta-analyses to operationalize specific definitions of the preventive treatments in CHR-P individuals. This is an essential step to address heterogeneity across different types of interventions and to characterize the specific nodes that were composing our network. We defined each treatment component as indicated in the following paragraphs.

#### Needs-based interventions (NBI)

CHR-P individuals enrolled in clinical trials are traditionally young people who are experiencing subtle symptoms and functional impairment (7) and who are therefore seeking help for their problems (8). Accordingly, it is felt unethical to randomize them to a pure placebo or “no treatment” condition (37). In this scenario it is also difficult to provide an exact definition of “treatment as usual,” because although treatment guidelines do exist (36), in reality treatment implementation is determined by local health service priorities, resources and configurations as well as availability of specialist training. We therefore decided on a pragmatic approach and adopted the operationalization of NBI provided by the founders of the CHR-P paradigm (38). This definition focuses on the symptoms and problems already presented by the help-seeking individual (39), and may encompass any of the following components: (a) needs-based supportive psychotherapy for problems with, for example, relationships, work or family; (b) case management for resolving issues with education, housing or employment; (c) brief family psychoeducation and general advice; (d) different types of medications other than antipsychotics; and (e) clinical monitoring alone or coupled with crisis management (38, 40).

#### Cognitive behavioral therapy, french and morrison protocol (CBT-F)

The CBT-F protocol (41), like the majority of CBT protocols, is grounded on the principles established by Beck (42). The intervention is problem-focused and time-limited, with treatment strategies selected based on the formulation of each patient's presenting problems but from a range of permissible, manualized strategies. Although each person's therapy will be tailored to their presenting needs, the core components include building engagement, collaborative goal-setting and formulation, normalizing psychotic-like experiences, evaluating core beliefs, and different types of behavioral experiments (41, 43).

#### Cognitive behavioral therapy, van der gaag protocol (CBT-V)

The protocol developed by van der Gaag et al. (44) is based on the French and Morrison protocol (41), which is then expanded by the addition of two novel components that target cognitive biases. The first additional component is education on dopamine system super-sensitivity and its relation to attenuated psychotic symptoms and exaggeration of cognitive biases, with the aim of normalizing aberrant perceptual experiences and reducing associated distress. The individual is taught how biases in cognition, such as selective attention, confirmation bias and jumping-to-conclusions contribute to the formation of delusions and paranoia (44). The second component involves exercises/behavioral experiments to correct these biases through examination of initial appraisals and testing of alternative explanations (45). Further aims of CBT-V include supporting school attendance and employment, improving relationships with friends and relatives, and if applicable, reducing cannabis use (44).

#### Integrated psychological interventions, bechdolf protocol (IPI)

The protocol developed by Bechdolf et al. (46) is a multi-component package of care. In addition to manualized and time-limited individual CBT-F (41), IPI also includes manualized group skills training, which focuses on scheduling and monitoring leisure activities, training in social skills, problem-solving and mastery of difficult situations, and developing “keeping well” strategies (46). The third component was computerized cognitive remediation to address thought and perception deficits (basic symptoms), and a final component included multi-family psychoeducation group sessions, which aimed to reduce interpersonal conflict and associated stress by helping family members better understand the CHR-P state (46, 47).

#### Family-focused therapy, miklowitz protocol (FFT)

The family-focused therapy (FFT) protocol by Miklowitz et al. (28) was originally developed for individuals with or at risk of bipolar disorder. The FFT was then adapted for CHR-P individuals, which has three broad stages. The first stage of FFT encompasses psychoeducation and the development of a patient-family prevention plan, which helps to increase understanding of the stressors contributing to attenuated positive and negative symptoms, while also detailing coping strategies and behavioral activation goals. The second stage focuses on enhancing constructive patient-family communication, and the third stage consists of improving problem-solving skills (28).

#### Psychopharmacological interventions

Pharmacological interventions included licensed medications, experimental pharmacotherapies as well as nutritional supplements.

#### Placebo

The placebo component was reserved for pharmacological placebos administered in the control arms of randomized controlled trials.

#### Node composition

We carefully identified the specific interventions (as listed above) for each arm of every study, which were then linearly combined to compose the precise treatment “nodes” of our network. As discussed above, this definition of nodes is an essential prerequisite for performing a robust NMA that can be of clinical relevance. Each pharmacological treatment was assigned to its own node, but different dosages of the same molecule were categorized within the same node. While placebo was initially considered as a separate node from NBI, after performing sensitivity analyses to explore the effect of pooling them together, we decided to combine them in the same node (see below for details).

### Search strategy and selection criteria

The first step of our literature search involved systematic electronic searches in the Web of Science (which includes Web of Science Core Collection, BIOSIS Citation Index, KCI-Korean Journal Database, MEDLINE, Russian Science Citation Index and SciELO Citation Index) and Ovid/ PsychINFO databases, the Cochrane Central Register of Controlled Trials and the NHS Centre for Reviews and Dissemination (CRD), using the following keywords: (risk OR prodromal OR prodrom^*^ OR ultra-high risk OR clinical high risk OR high risk OR genetic high risk OR at risk mental state OR risk of progression OR progression to first-episode OR prodromally symptomatic OR basic symptoms) AND (psychosis) AND (RCT OR randomized controlled trial OR placebo controlled trial OR trial). The searches were conducted up to 18th July 2017 and no language restrictions were applied. In a second step, we used Scopus/Web of Science to screen the reference lists of articles identified in the previous step and those of existing systematic reviews and meta-analyses. For comprehensiveness, we also searched the reference lists of relevant clinical guidelines. In a third step, we looked for published and unpublished material in relevant conference proceedings, trial registries (e.g., https://clinicaltrials.gov) or regulatory agencies. The OpenGrey database (http://www.opengrey.eu) was used to identify unpublished material from the gray literature.

The above search strategies led us to identifying potential abstracts of interest. The abstracts were then screened for potential inclusion and those that survived this initial filter were downloaded as full-text articles. These were then carefully inspected against the full inclusion and exclusion criteria which are described below.

In line with the PRISMA guidance, two independent researchers conducted the literature search, study selection and data extraction (48). During the above steps, disagreement between extractors was addressed through discussion with a third researcher until consensus was obtained. We defined the specific inclusion and exclusion criteria to ensure that the population represented in the final database would be broadly representative of the target CHR-P population as a whole (49).

Our inclusion criteria were (a) being an original article, abstract or pilot study; (b) being a randomized controlled trial (including cluster randomized trials, but excluding cross-over studies); (c) being designed as blinded (either single- or double-blind); (d) being conducted in CHR-P individuals with CHR-P criteria ascertained through the use of internationally validated psychometric assessments, i.e., the Comprehensive Assessment of At-Risk Mental States (CAARMS) (6), the Structured Interview for Psychosis-risk Syndromes (SIPS) (50), the Positive and Negative Syndrome Scale (PANSS) (51), the Brief Psychiatric Rating Scale (BPRS) (52), or the Early Recognition Inventory (ERIraos) (53); (e) comparing specific preventive interventions as defined in the sections above; (f) providing sufficient data to perform meta-analytic computation; (g) providing a sample size of 10 or greater (54).

Our exclusion criteria were defined as (a) being a review or reporting non-original data; (b) lacking at least two compared groups, such as open-label trials in a single group of CHR-P patients exposed to treatment; (c) investigating patient samples affected with an established first-episode psychosis or any at-risk group other than CHR-P samples; (d) lacking sufficient data needed to perform the essential meta-analytical computations; (e) design lacking proper randomization, such as quasi-randomization or observational naturalistic studies - however, studies that were initially conceived and designed as blinded but could not maintain blinding during follow-up (e.g., for psychological interventions) were not excluded; (f) including a sample size smaller than 10 (i.e., *N* = 9 or less); (g) presenting overlapping data (i.e., for the same outcome at the same time point as data that was already included)-in the case of overlapping data/samples, we preferred the data relating to the largest sample size.

### Outcome measures and data extraction

Due to the variable effect of time on clinical outcomes in these samples (9, 55), analyses for time-dependent outcomes were conducted. The primary outcome (efficacy) was the level of attenuated positive psychotic symptoms at follow-up, indexed by the relevant subscales of validated assessments, such as the PANSS, CAARMS, BPRS and SIPS. For each arm of every study, we extracted the mean and standard deviation (SD) of these scores at 6 and 12 month follow-up time points. Where studies did not report sufficient data to extract the primary outcome, we used DigitizeIt software (http://www.digitizeit.de/) to extract data presented graphically (means and 95% confidence intervals (CIs) for each follow-up time point). When necessary, SDs were back-calculated using standard formulae. If none of the aforementioned were available, we estimated follow-up data using information available from the published paper and using assumptions established in previous literature. Sample sizes were based on the numbers randomized to each arm.

A high benefit-to-harm ratio is essential when adopting preventive strategies that may lead to the unnecessary treatment of false positives. We therefore selected the acceptability of interventions (discontinuation due to any cause) as our secondary outcome measure. In line with previous authoritative publications, we defined the acceptability of interventions as the number of participants who dropped out of each arm for any reason following randomization, over those randomized at baseline (56–58).

In order to describe our population, assess the transitivity assumption (see below), address the risk of bias and conduct meta-regression analyses, we also extracted details on the first author and year of publication of each trial, country where the trial was conducted, types of outcomes reported, definitions of intervention and control arms (in line with the treatment components described above), trial design, risk of bias assessment, duration of each intervention and follow-up, sample size, mean age, percent male, and the psychometric CHR-P instrument used to ascertain attenuated positive psychotic symptoms.

#### Risk of bias

The assessment of bias is of paramount importance for rigorously interpreting the results of evidence synthesis studies and for testing their robustness. We used the Cochrane Risk of Bias tool (59) to classify the risk of bias in each study using *a priori* defined criteria. Using these standardized criteria, we evaluated whether each trial was at high, low or unclear risk of bias across six specific domains. These included random sequence generation, allocation concealment, blinding of participants and study personnel, blinding of outcome assessments, incomplete outcome data, and selective outcome reporting. Once these domains were assessed, the Cochrane Risk of Bias tool allowed production of an overall risk of bias classification of high, low or unclear. The overall rating of low risk was assigned when none of the six domains were found to be at high risk and if three or less domains were found to be at unclear risk. The overall rating of moderate risk was assigned when one domain was found to be at high risk; or no domains were found to be at high risk but four or more were found to be at unclear risk. In all other cases, the trial was classified as having an overall high risk of bias (60).

### Statistical analysis

#### Network meta-analysis

Frequentist NMAs were conducted for both primary (attenuated positive psychotic symptoms) and secondary (acceptability) outcomes at 6 and 12 months using the *network* package in STATA (version SE 14.2). Effect sizes for the primary outcome were calculated and reported as the standardized mean difference Hedges' adjusted *g* (SMD) and 95% CIs in mean follow-up scores between two compared interventions, using the pooled SD at follow-up (61). Follow-up data are considered preferable when measuring continuous outcomes that are difficult to measure (62). Effect sizes for the secondary outcome were reported as odds ratio (OR) and 95% CIs. We first constructed network plots -for each outcome- to ensure that the geometry of the networks were sufficiently connected (63, 64). We then performed a NMA assuming consistency and a common heterogeneity across all comparisons in the network. This allowed us to derive a single summary treatment effect (SMD for attenuated positive psychotic symptoms; OR for acceptability) for every possible pairwise comparison of treatments. This summary effect draws on all evidence from the network of trials, including direct and indirect evidence. Correlations in effect sizes induced by multi-arm trials were accounted for (63, 65). The resulting relative SMDs or ORs with 95% CIs for each pair of treatments were reported in league tables (66). Statistical significance was set at *p* < 0.05.

When performing NMA it is possible to rank an outcome of interest using the Surface Under the Cumulative RAnking curve (SUCRA) procedure. Such an approach allows integration of both the location and the variance of any relative effect on the outcome of interest (67). In simple terms, the SUCRA procedure summarizes the overall ranking of each intervention through a single number ranging from 0 to 100% (68). In this manuscript, the higher the SUCRA value, the higher the likelihood that an intervention will be in the top rank, and vice versa (68). In line with our objective, we performed cluster ranking (63, 67) of the SUCRA values for attenuated positive psychotic symptoms and acceptability (at 6 and 12 months, separately) and presented the results in two-dimensional plots (64). These plots aid visualization of the relative balance between a treatment's ranking across different outcomes, and show the clustering of treatments into meaningful groups as determined by hierarchical cluster analysis (63, 64).

Consistency in a network refers to the equivalence of direct and indirect estimates of the same treatment comparison pairs, and can be investigated in each closed loop of evidence (66). We assessed this assumption by calculating an inconsistency factor along with 95% CIs (truncated at 0) and associated *p*-values for each closed loop of the primary outcome (63). Inconsistency was defined as disagreement between direct and indirect evidence, with 95% CIs for inconsistency factors excluding zero. Because the loop-specific approach focuses on local inconsistency and has low power, we also tested a full design-by-treatment model (69) for the primary outcome to evaluate global inconsistency. This entailed performing a NMA under the inconsistency model and using the χ^2^-test to estimate the statistical significance of all possible inconsistencies in the networks (70).

Transitivity was examined by assessing the distributions of potential effect modifiers across comparisons in the networks. These effect modifiers encompassed the following items: percent male, age, percent exposed to antipsychotic medications at baseline, type of blinding and publication year.

The presence of small-study effects was assessed by visual inspection of comparison-adjusted funnel plots (71). In this analysis we used NBI (or when not available, CBT-F + NBI) as the reference. We assumed that small-study effects, if present, would be expected to exaggerate the effectiveness of the “active” (or newer/experimental) treatment, rather than NBI or CBT-F + NBI, which currently represent the most widely implemented interventions for this patient group.

#### Complementary analyses

Sensitivity analyses were performed to address the impact of study quality and our data analysis strategy. Specifically, we repeated the NMA analyses on attenuated positive psychotic symptoms using only: (a) studies with a low risk of bias for the blinding of attenuated positive psychotic symptom assessments; (b) studies whose meta-analytical data (i.e., mean and SD of attenuated positive psychotic symptoms) were not estimated using assumptions established in previous literature; and (c) published trials only (i.e., excluding conference proceedings). In addition, we repeated the NMA after pooling NBI and placebo nodes and after pooling different types of antipsychotic molecules. To ensure that the use of follow-up scores did not unduly influence our results, we repeated the analyses using SMD calculated from change score and pooled baseline SD, which is recommended when a full ANCOVA model is not feasible (62). Furthermore, we complemented the sensitivity analyses through network meta-regression analyses. These were planned only when substantial heterogeneity was observed and when at least 10 independent trials were available (72) for each outcome of interest. These meta-regressions were planned to investigate the potential impact of the different CHR-P psychometric instruments used for measuring attenuated positive psychotic symptoms (34).

For the primary outcome, we also conducted conventional pairwise meta-analyses (random effects model) of every direct treatment comparison using the *metan* package in STATA. The random effects meta-analyses were stratified by (a) follow-up time (6 or 12 months), and (b) pairwise intervention comparisons (i.e., each type of treatment vs. its control was treated as a meta-analysis, no overall summary effect computed across comparisons of different treatments). The resulting meta-analytic SMDs together with 95% CIs and measures of heterogeneity (I^2^) were calculated and presented in tables. When pairwise groups had more than three contributing studies, we performed leave-one-out sensitivity analyses to explore the robustness of the results to influential individual studies.

## Results

### Characteristics of trials and patients

**Figure 1:**
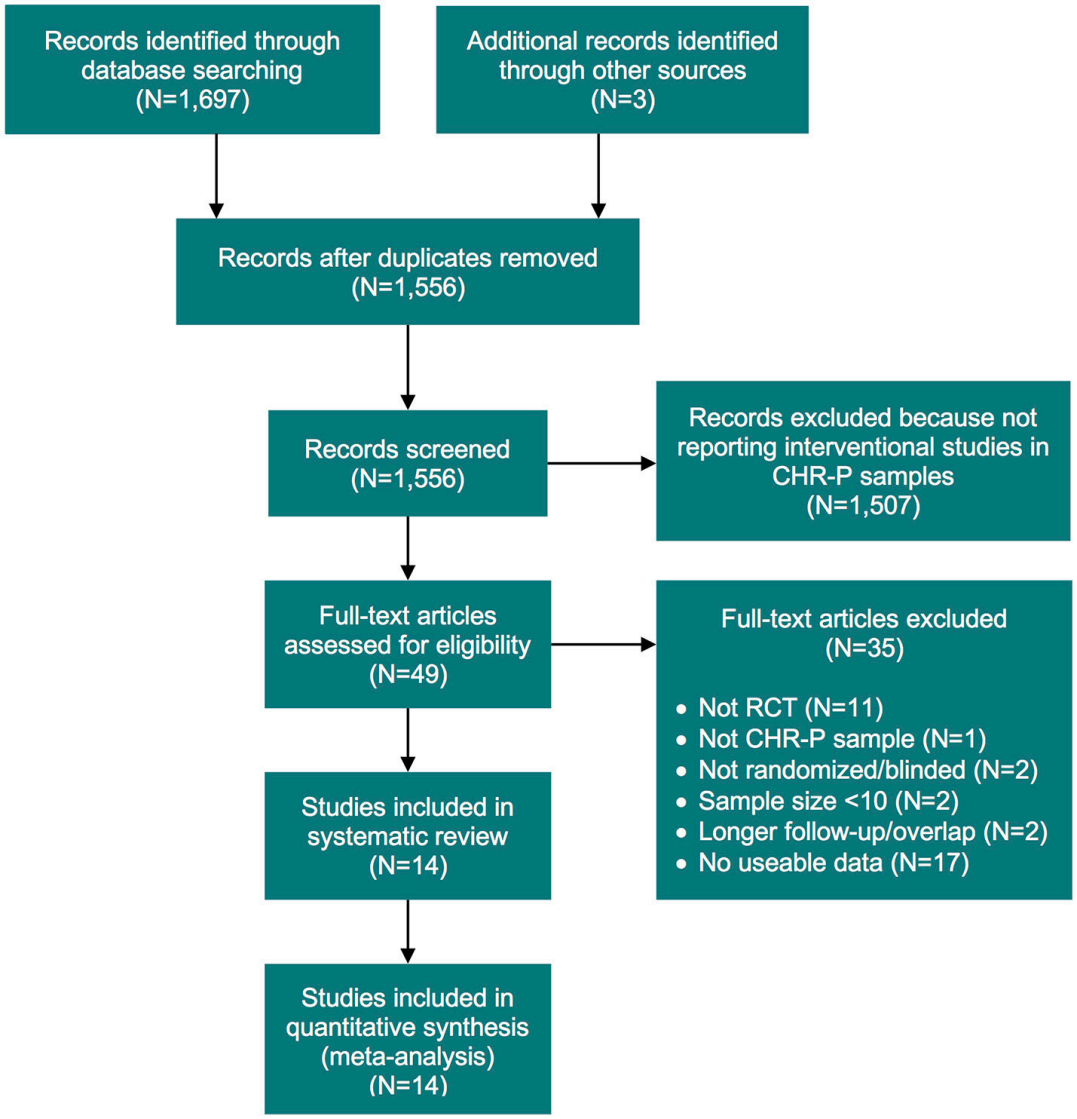
PRISMA flow chart of the study selection process. CHR-P, clinical high risk for psychosis; RCT, randomized controlled trial.

**Figure 2:**
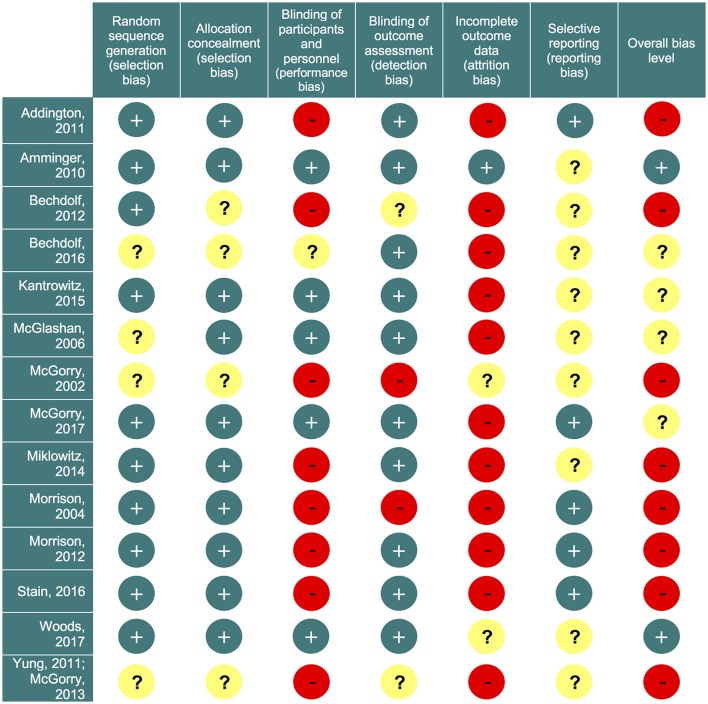
Risk of bias summary. Review authors' judgements about each risk of bias item for each included study. Green circles (+) indicate low risk of bias; yellow circles (?) indicate unclear risk; red circles (−) indicate high risk of bias.

Our initial literature search identified 1,556 references. However, most of them did not report on RCTs in CHR-P individuals. As indicated in Figure 1, 49 of them were eventually downloaded and fully inspected against the inclusion and exclusion criteria, which resulted in a final sample of 14 studies. We found only three, three, two and two trials reporting the outcome data of interest at 18, 24, 36, and >36 month time points, respectively. Consequently, the current meta-analysis focuses only on the 6 and 12 month time points. The 14 studies used in the analyses contributed data on a total of 1707 patients, with a mean age of 20.1 ± 2.9 years, and of whom 57% were male (Table 1). The mean sample size was 122 (range 44–304). Five studies were conducted in North America, five in Europe, three in Australia and one was multi-national. Two trials adopted a three-arm design while all of the others employed a two-arm design. Two of the included studies were identified from conference proceedings and gray literature/clinical trial databases (24, 30). Two studies had a treatment duration of <6 months, eight of 6 months, and four of 12 months. Three of the 12 trials that reported enough information to identify the source of sponsorship or funding acknowledged pharmaceutical company involvement. The SIPS was the most common assessment used for measuring attenuated positive psychotic symptoms (*N* = 6). Only two studies had an overall low risk of bias (30, 40), four had unclear risk (24, 26, 27, 73) and the remaining eight had high risk; the full risk of bias assessment is presented in Figure 2.

**Table 1:** Characteristics of included studies.

**Study**	**Study arms (N)**	**Total N**	**Network inclusion**	**Treatment duration (months)**	**Follow-up time points (months)**	**% male**	**Mean age**	**Measure of symptoms**	**Study design**	**Country**	**% exposed to antipsychotics at baseline**
Addington et al. (37)	CBT-F + NBI [27] NBI [24]	51	6, 12	6	6, 12, 18	71	20.9	SIPS	SB-RCT	Canada	0
Amminger et al. (40)	Omega-3 + NBI [41] NBI [40]	81	6, 12	3	6, 12, 84	33	16.4	PANSS	DB-RCT	Austria	0
Bechdolf et al. (46, 75)	IPI [63] NBI [65]	128	12	12	6, 12, 18, 24	63	26.0	PANSS	SB-RCT	Germany	0
Bechdolf et al. (24)	ARI + NBI [96] NBI [55] CBT-F + NBI [129]	280	6, 12	12	6, 12	66	24.4	SIPS	SB-RCT	Germany	3.4
Kantrowitz et al. (26)	D-serine + NBI [20] NBI [24]	44	6	4	4	66	19.4	SIPS	DB-RCT	US	11.4
McGlashan et al. (73)	OLA + NBI [31] NBI [29]	60	12	12	12, 24	65	17.7	SIPS	DB-RCT	US, Canada	10
McGorry et al. (38)	RIS + CBT-F + NBI [31] NBI [28]	59	6, 12	6	6, 12, 36–48	58	20.0	BPRS	SB-RCT	Australia	0
McGorry et al. (27)	Omega-3 + NBI [153] NBI [151]	304	6, 12	6	6, 12	46	19.2	BPRS	DB-RCT	Multi-national	0
Miklowitz et al. (28)	FFT + NBI [66] NBI [63]	129	6	6	6	57	17.4	SIPS	SB-RCT	US, Canada	20.9
Morrison et al. (43)	CBT-F + NBI [37] NBI [23]	60	12	6	6, 12, 36	69	22.0	PANSS	SB-RCT	UK	0
Morrison et al. (74)	CBT-F + NBI [144] NBI [144]	288	6, 12	6	6, 12, 18, 24	63	20.7	CAARMS	SB-RCT	UK	0
Stain et al. (29)	CBT-F + NBI [30] NBI [27]	57	6, 12	6	6, 12	40	16.3	CAARMS	SB-RCT	Australia	0
Woods et al. (30, 31)	ZIP + NBI [24] NBI [27]	51	6	6	6	64	22.3	SIPS	DB-RCT	US	0
Yung et al. (76) and McGorry et al. (77)	CBT-F + NBI [44] RIS + CBT-F + NBI [43] NBI [28]	115	6, 12	12	6, 12	39	18.1	BPRS	SB-RCT	Australia	0

*CBT-F, cognitive behavioral therapy (French and Morrison protocol); NBI, eeds-based interventions (including placebo); IPI, integrated psychological interventions; ARI, aripiprazole; OLA, olanzapine; RIS, risperidone; FFT, family-focused therapy; ZIP, ziprasidone; SIPS, Structured Interview for Psychosis-risk Syndromes; PANSS, Positive and Negative Syndrome Scale; BPRS, Brief Psychiatric Rating Scale; CAARMS, Comprehensive Assessment of At-Risk Mental States; SB-RCT, single-blind randomized controlled trial; DB-RCT, double-blind randomized controlled trial*.

**Figure 3:**
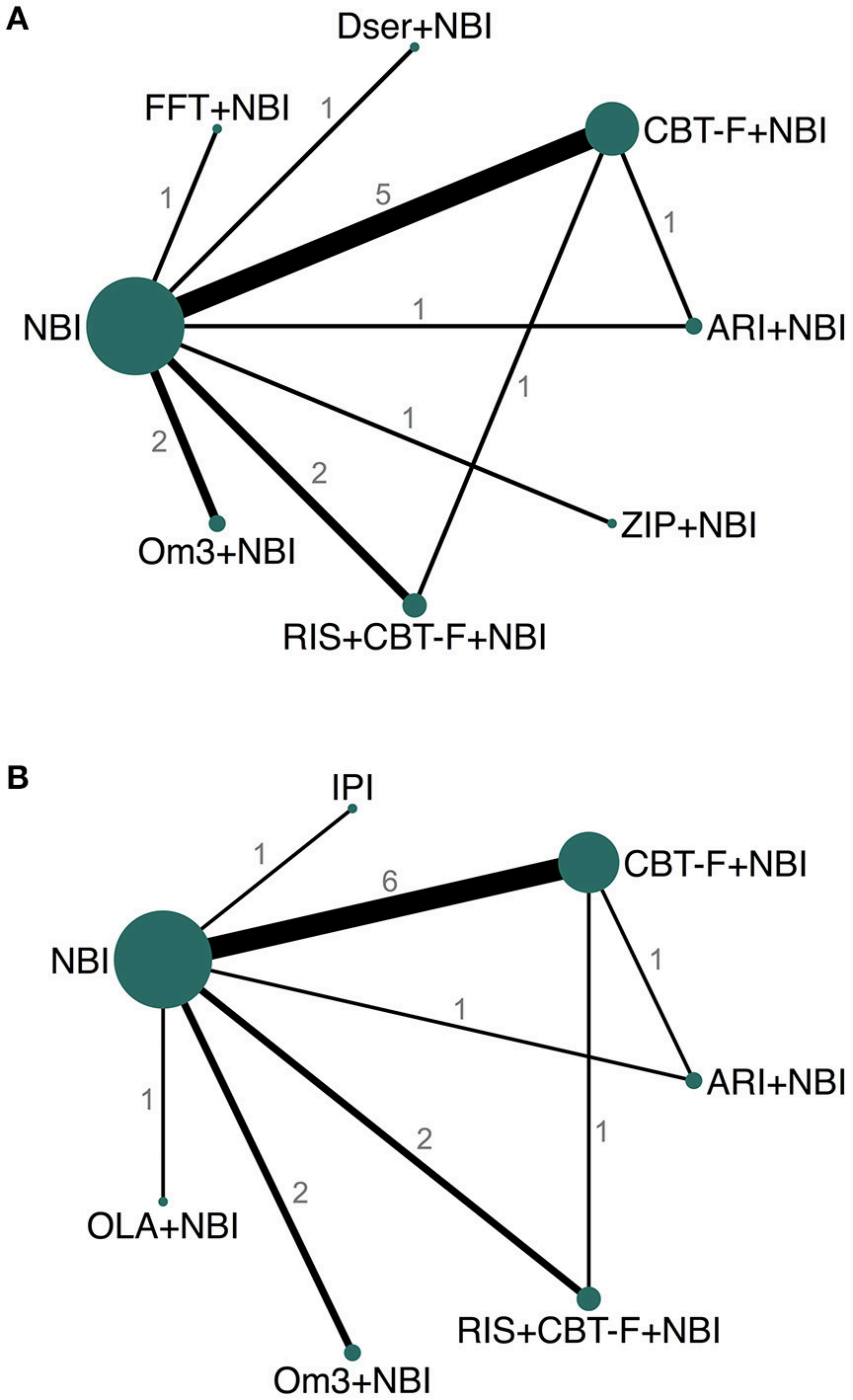
Network plots of direct comparisons at 6 **(A)** and 12 **(B)** months. The width of the lines is proportional to the number of trials comparing each pair of treatments and the size of each node is proportional to the number of studies testing the specific treatment. Numbers represent the number of studies contributing data to each comparison. ARI, aripiprazole; NBI, needs-based interventions (including placebo); CBT-F, cognitive behavioral therapy (French and Morrison protocol); Dser, D-serine; FFT, family-focused therapy; Om3, omega-3 fatty acids; RIS, risperidone; ZIP, ziprasidone; IPI, integrated psychological interventions; OLA, olanzapine.

For the primary outcome, eight studies provided data for both 6 and 12 month networks, three only provided data for 6 months, and another 3 only for 12 months, resulting in 11 studies contributing data for the 6 month analysis, and 11 to the 12 month analysis. For the 6 month analysis, 11 studies (*N* = 1459) provided data on 15 direct comparisons between 8 different treatment nodes (Figure 3A). For the 12 month analysis, 11 studies (*N* = 1483) provided data on 15 direct comparisons between 7 different treatment nodes (Figure 3B).

At 6 months, seven studies provided the required follow-up symptom data directly or indirectly, two provided means and SD graphically (28, 40), and for two studies symptom data were estimated on the basis of available data and assumptions established in previous literature (27, 30). At 12 months, nine studies provided the required data directly or indirectly, one provided data graphically (40), and for one study symptom data were estimated on the basis of available data and assumptions established in previous literature (27).

All studies, except one (38), provided data for the secondary outcome (acceptability) at both 6 and 12 months. Network plots for the acceptability outcome were the same as those for the primary outcome (Figure 3).

### Pairwise meta-analysis

Pairwise meta-analysis results for the primary outcome are presented in Table 2. Only three pairwise intervention vs. control groups had two or more studies: CBT-F + NBI vs. NBI; omega-3 + NBI vs. NBI; and risperidone + CBT-F + NBI vs. NBI. The remaining pairwise intervention vs. control groups were composed of single studies.

**Table 2:** Pairwise meta-analytic results for attenuated psychotic symptoms at 6 and 12 months.

**Time point; months**	**Treatment condition**	**Control condition**	**Number of studies (references)**	**Total N**		**SMD (*g)***	**Lower 95%CI**	**Upper 95%CI**	**Heterogeneity**		
				**Treatment**	**Control**				**I^2^**	**Q**	**P**
6	ARI + NBI	NBI	1 (24)	96	55	−0.22	−0.56	0.11	–	0.00	–
	ARI + NBI	CBT-F+NBI	1 (24)	96	129	−0.06	−0.33	0.20	–	0.00	–
	CBT-F + NBI	NBI	5 (24, 29, 37, 74, 76)	374	278	−0.06	−0.26	0.13	24.2	5.27	0.26
	Dser + NBI	NBI	1 (26)	20	24	−0.10	−0.70	0.49	–	0.00	–
	FFT + NBI	NBI	1 (28)	66	63	−0.41	−0.76	−0.06	–	0.00	–
	Om3 + NBI	NBI	2 (27, 40)	194	191	−0.48	−1.62	0.67	94.8	19.4	<0.001
	RIS + CBT-F + NBI	CBT-F+NBI	1 (76)	43	44	0.37	−0.05	0.80	–	0.00	–
	RIS + CBT-F + NBI	NBI	2 (38, 76)	74	56	0.02	−0.33	0.37	0.00	0.72	0.40
	ZIP + NBI	NBI	1 (30)	24	27	−1.10	−1.69	−0.50	–	0.00	–
12	ARI + NBI	NBI	1 (24)	96	55	−0.22	−0.55	0.12	–	0.00	–
	ARI + NBI	CBT-F+NBI	1 (24)	96	129	−0.08	−0.34	0.18	–	0.00	–
	CBT-F + NBI	NBI	6 (24, 29, 37, 43, 74, 77)	411	301	**−0.22**	**−0.37**	**−0.07**	0.00	3.27	0.66
	IPI	NBI	1 (46)	63	65	0.20	−0.15	0.54	–	0.00	–
	OLA + NBI	NBI	1 (73)	31	29	−0.53	−1.05	−0.02	–	0.00	–
	Om3 + NBI	NBI	2 (27, 40)	194	191	−0.38	−1.38	0.63	93.5	15.49	<0.001
	RIS + CBT-F + NBI	CBT-F+NBI	1 (77)	43	44	−0.07	−0.49	0.35	–	0.00	–
	RIS + CBT-F + NBI	NBI	2 (38, 77)	74	56	0.00	−0.38	0.38	16.2	1.19	0.28

*Underlined bold text within the SMD and 95% CI columns indicates statistically significant meta-analytic treatment effect. SMD below 0 favors the given treatment condition. ARI, aripiprazole; NBI, needs-based interventions (including placebo); CBT-F, cognitive behavioral therapy (French & Morrison protocol); Dser, D-serine; FFT, family-focused therapy; Om3, omega-3 fatty acids; RIS, risperidone; ZIP, ziprasidone; IPI, integrated psychological interventions; OLA, olanzapine. Dashes (–) indicate no heterogeneity estimate due to having only one contributing study (and thus cannot be considered a true meta-analytic result)*.

At 6 months, there was no significant difference between CBT-F + NBI vs. NBI alone (SMD = −0.06, 95% CI −0.26 to 0.13; 5 studies, *N* = 652). However, there was meta-analytical evidence of a greater reduction in attenuated positive psychotic symptoms in CBT-F + NBI vs. NBI alone at 12 months (SMD = −0.22, 95% CI −0.37 to −0.07; 6 studies, *N* = 712). Leave-one-out sensitivity analyses showed that this effect was dependent on the presence of one study (74). When this study was removed, the combined effect at 12 months became non-significant (SMD = −0.12, 95% CI −0.32 to 0.08; 5 studies, *N* = 424). The non-significant summary effect at 6 months did not become significant throughout any iteration of the leave-one-out analyses.

Two studies compared omega-3 + NBI to NBI alone, but both 6 and 12 month summary effect estimates were not significant (6 month SMD = −0.48, 95% CI −1.62 to 0.67, 2 studies, *N* = 385; 12 month SMD = −0.38, 95% CI −1.38 to 0.63, 2 studies, *N* = 385). Significant heterogeneity was detected between these two studies at both 6 (*I*^2^ = 95%, *p* < 0.001) and 12 (*I*^2^ = 94%, *p* < 0.001) month time points. Statistical investigation of potential sources of heterogeneity using meta-regression was precluded by the limited number of studies.

Combined therapy with risperidone + CBT-F + NBI was not significantly different from NBI alone at either 6 (SMD = 0.02, 95% CI −0.33 to 0.37, 2 studies, *N* = 130) or 12 months (SMD = 0.00, 95% CI −0.38 to 0.38, 2 studies, *N* = 130). While available data on all further pairwise treatments vs. controls are listed in Table 2 for completeness, they represent single studies and thus cannot be considered meta-analytic results.

### Network meta-analysis – effect on attenuated positive psychotic symptoms

Results of the NMA are presented in Tables 3, 4. At 6 months, ziprasidone + NBI was found to be significantly more effective at reducing attenuated positive psychotic symptoms compared to NBI alone (SMD = −1.10, 95% CI −2.04 to −0.15); compared to CBT-F + NBI (SMD = −1.03, 95% CI −2.05 to −0.01); and compared to risperidone + CBT-F + NBI (SMD = −1.18, 95% CI −2.29 to −0.07). There were no other significant effects of any one intervention over any others (Table 3). Using NBI as a comparator, the relative treatment effect estimates (all SMD < 0 favor the given treatment) at 6 months were: ziprasidone + NBI (SMD = −1.10, 95% CI −2.04 to −0.15); omega-3 + NBI (SMD = −0.42, 95% CI −1.01 to 0.16); aripiprazole + NBI (SMD = −0.18, 95% CI −0.90 to 0.53); family-focused therapy + NBI (SMD = −0.41, 95% CI −1.22 to 0.41); CBT-F + NBI (SMD = −0.07, 95% CI −0.44 to 0.31); D-serine + NBI (SMD = −0.10, 95% CI −1.05 to 0.84); and risperidone + CBT-F + NBI (SMD = 0.08, 95% CI −0.50 to 0.67).

**Table 3:**
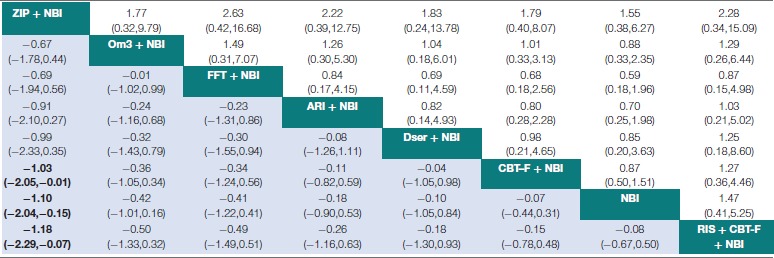
Network meta-analytic relative treatment effects for efficacy and acceptability at 6 months.

*Comparisons between treatments should be read from left to right, and the estimate is in the cell in common between the column-defining treatment and the row-defining treatment. For the primary outcome (attenuated positive psychotic symptoms) estimates, results are SMD (95% CI), where SMD below 0 favors the column-defined treatment. For acceptability, results are OR (95% CI), where OR < 1 favors the row-defined treatment. Significant results are in bold. The order of treatments in the diagonal is arbitrary and does not reflect ranking. ZIP, ziprasidone; NBI, needs-based interventions (including placebo); Om3, omega-3 fatty acids; FFT, family-focused therapy; ARI, aripiprazole; Dser, D-serine; CBT-F, cognitive behavioral therapy (French & Morrison protocol); RIS, risperidone*.

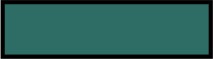
*Comparison*.

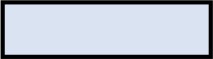
*Efficacy (attenuated psychotic symptoms; SMD [95% CI])*.

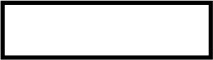
*Acceptability (dropout; OR [95% CI])*.

**Table 4:**
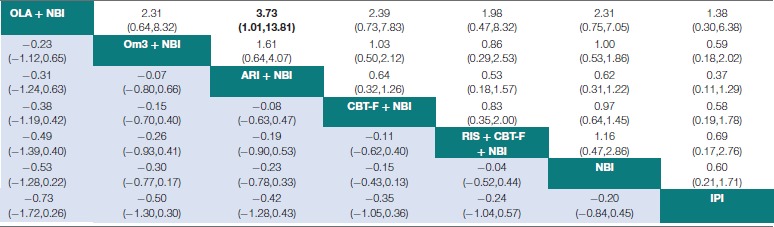
Network meta-analytic relative treatment effects for efficacy and acceptability at 12 months.

*Comparisons between treatments should be read from left to right, and the estimate is in the cell in common between the column-defining treatment and the row-defining treatment. For the primary outcome (attenuated positive psychotic symptoms) estimates, results are SMD (95% CI), where SMD below 0 favors the column-defined treatment. For acceptability, results are OR (95% CI), where OR < 1 favors the row-defined treatment. Significant results are in bold. The order of treatments in the diagonal is arbitrary and does not reflect ranking. OLA, olanzapine; NBI, needs-based interventions (including placebo); Om3, omega-3 fatty acids; ARI, aripiprazole; CBT-F, cognitive behavioral therapy (French & Morrison protocol); RIS, risperidone; IPI, integrated psychological interventions*.

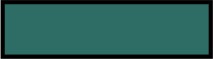
*Comparison*.

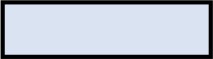
*Efficacy (attenuated psychotic symptoms; SMD [95% CI])*.

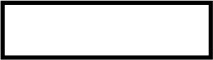
*Acceptability (dropout; OR [95% CI])*.

At 12 months, there was no evidence that any one intervention was superior over any others, with all 95% CIs crossing zero (Table 4). Using NBI as a comparator, the relative treatment effect estimates (all SMD < 0 favor the given treatment) at 12 months were: olanzapine + NBI (SMD = −0.53, 95% CI −1.28 to 0.22); omega-3 + NBI (SMD = −0.30, 95% CI −0.77 to 0.17); aripiprazole + NBI (SMD = −0.23, 95% CI −0.78 to 0.33); CBT-F + NBI (SMD = −0.15, 95% CI −0.43 to 0.13); risperidone + CBT-F + NBI (SMD = −0.04, 95% CI −0.52 to 0.44); and integrated psychological interventions (SMD = 0.20, 95% CI −0.45 to 0.84).

#### Inconsistency and small-study effects

There was no statistically significant inconsistency in the 6 or 12 month networks. The 95% CIs for all inconsistency factors were compatible with zero inconsistency. However, it is important to note that only two loops were available at both 6 and 12 months, which may have limited our ability to detect inconsistency. When we used the design-by-treatment interaction model, there was no evidence for significant inconsistency in the 6 (*p* = 0.92) or 12 month (*p* = 0.92) networks.

Visual inspection of comparison-adjusted funnel plots suggested no clear small-study effects (publication biases), with a regression line almost flat at 6 months (Figure S1A in Supplementary Material) and completely flat at 12 months (Figure S1B in Supplementary Material).

#### Sensitivity analyses for NMA of primary outcome

We tested the robustness of the core NMA findings (that ziprasidone + NBI is superior to NBI alone, CBT-F + NBI, and risperidone + CBT-F + NBI at 6 months) through various sensitivity analyses. At 6 months, two studies (27, 30) were based on estimated follow-up data, one of which was the single ziprasidone + NBI vs. NBI study (30). Repeating the analyses after removal of the latter study (30) inherently meant that there was now no ziprasidone + NBI node and all estimates were non-significant. Removal of the other study -by McGorry et al (27)- did not affect the current results at 6 or 12 months; however, one change of note is that at 12 months, CBT-F + NBI became significantly more effective than NBI, which is interesting in light of the pairwise significance of CBT-F + NBI vs. NBI at 12 months, and lack thereof in the main NMA analyses.

**Figure 4:**
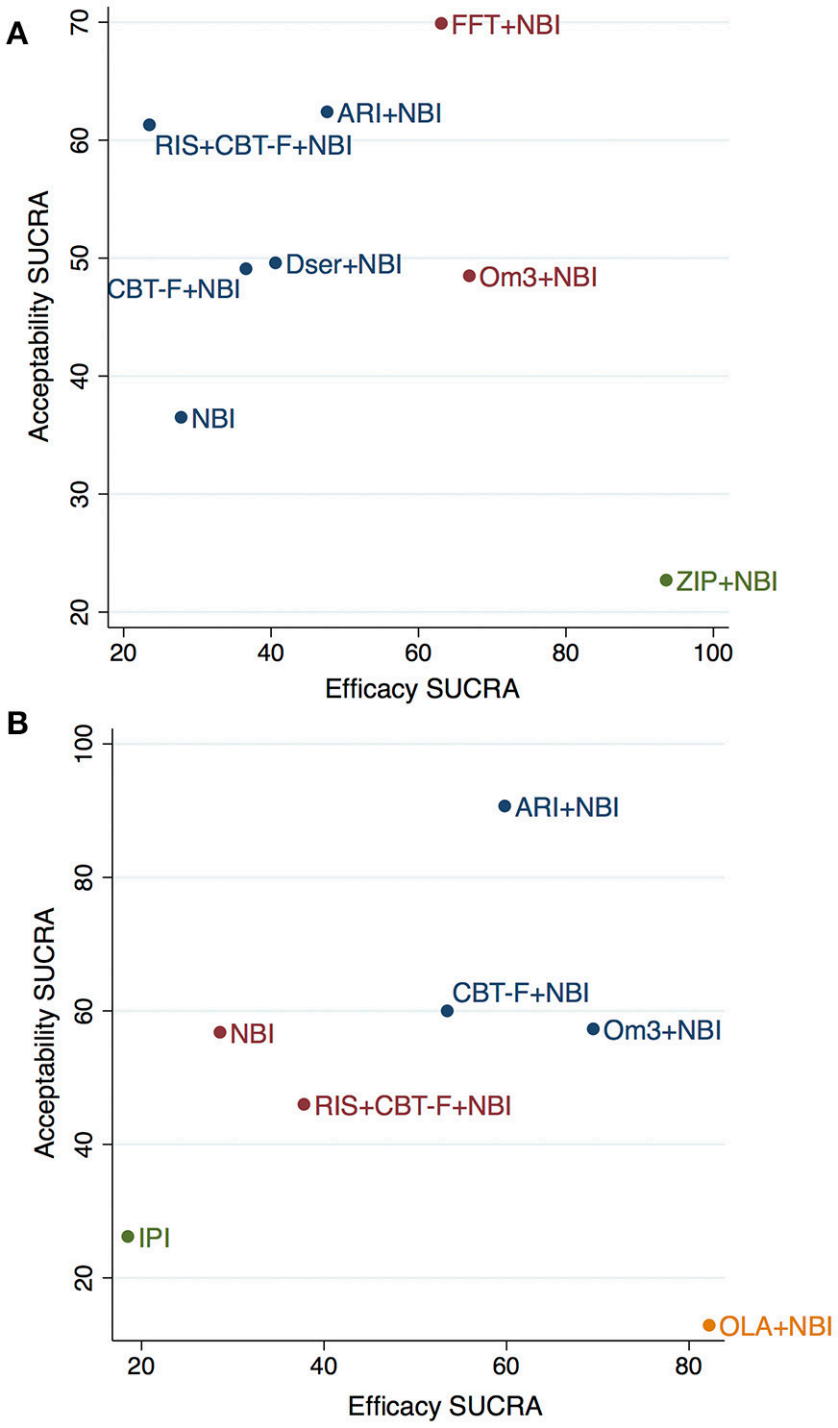
Cluster-ranking of treatments according to SUCRA values for efficacy and acceptability at 6 **(A)** and 12 **(B)** months. Colors reflect treatments belonging to the same cluster. Please note that although the treatments have been ranked, there is no statistical difference between them -with the exception of ziprasidone+NBI for efficacy- (see Tables 3,4 for further details). ARI, aripiprazole; NBI, needs-based interventions (including placebo); CBT-F, cognitive behavioral therapy (French and Morrison protocol); Dser, D-serine; FFT, family-focused therapy; Om3, omega-3 fatty acids; RIS, risperidone; ZIP, ziprasidone; IPI, integrated psychological interventions; OLA, olanzapine.

The ziprasidone + NBI results were not robust to the removal of two studies (38, 76) at high or unclear risk of bias for the blinding of outcome assessments, which resulted in only the ziprasidone + NBI vs. NBI comparison remaining significant. Repeating the analyses after removing unpublished studies (24, 30), pooling together different antipsychotic molecules, and using change scores instead of follow-up scores all abolished the ziprasidone + NBI results. Repeating the analyses treating NBI + placebo as a separate node to NBI had some effect on the NMA estimates at 6 months (ziprasidone + NBI was now superior only to NBI + placebo) and had no effect at 12 months; we therefore used the pooled NBI + placebo in the main analysis (Tables 1–4, Figures 3, 4). There were too few studies to allow robust meta-regression analyses on the type of instruments used to measure attenuated positive psychotic symptoms.

### Network meta-analysis – effect on acceptability

There were no significant differences in acceptability between any treatments at 6 months (Table 3). At 12 months, aripiprazole + NBI was significantly more acceptable than olanzapine + NBI (*OR* = 3.73; 95% CI 1.01 to 13.81). There were no further significant differences at 12 months (Table 4). However, at both time points, the 95% CIs for the comparisons were often very wide, indicating substantial imprecision in the estimates.

### Network meta-analysis – cluster ranking for attenuated positive psychotic symptoms and acceptability

The cluster ranking plots of SUCRA values for attenuated positive psychotic symptoms (efficacy) and acceptability are illustrated in Figure 4A (for 6 months) and Figure 4B (for 12 months). However, it should be noted that although the treatments were cluster ranked, there was no statistically significant difference between any treatments (with the exception of ziprasidone + NBI) in the main network meta-analysis results (see Tables 3, 4 for details).

Three distinct clusters were found in the cluster ranking at 6 months (Figure 4A). Notably, while ziprasidone + NBI had the highest SUCRA for efficacy (94%), it was also the most poorly tolerated, having the lowest SUCRA value for acceptability (23%). In a second cluster, omega-3 + NBI and family-focused therapy + NBI had similar SUCRA scores for efficacy (67% and 63%, respectively), however, they differed markedly in their SUCRA for acceptability; family-focused therapy + NBI had the highest acceptability SUCRA of all treatments (70%), while that of omega-3 + NBI was mid-range (49%). The third cluster comprised the remaining treatments, whose SUCRA values for efficacy were all below 50%, but whose acceptability SUCRAs varied from 62% for aripiprazole + NBI, to 37% (the worst) for NBI.

At 12 months, four distinct clusters were found (Figure 4B). Similar to above, while olanzapine + NBI was ranked highest of all treatments for efficacy SUCRA (82%), it also scored worst for acceptability (13%). A second cluster, with a more balanced profile of efficacy and acceptability SUCRA values, comprised aripiprazole + NBI, omega-3 + NBI and CBT-F + NBI. Of these, omega-3 + NBI had the highest SUCRA value in terms of efficacy (70%) but lower acceptability (57%), aripiprazole + NBI had slightly lower efficacy (60%) but the highest acceptability of all treatments (91%), and CBT-F + NBI had mid-range values for both outcomes. NBI and risperidone + CBT-F + NBI were found in a third, intermediate cluster with low mid-range SUCRA values. The final cluster was composed of integrated psychological interventions, with SUCRA values of 19% and 26% for efficacy and acceptability, respectively.

## Discussion

To the best of our knowledge, this is the first network meta-analysis to have explored the effect of preventive treatments on attenuated positive psychotic symptoms in CHR-P individuals. Focusing exclusively on RCTs to minimize selection biases, we included a total of 14 non-overlapping studies, for a total database of 1,707 CHR-P individuals, representing the largest evidence synthesis of this topic to date. By using the most updated evidence we defined two networks at 6 and 12 months, on which we performed the core analyses. These two networks included 8 and 7 nodes, respectively. There were not enough studies to generate networks beyond these time points. Overall, our network meta-analyses indicated no robust evidence of superior efficacy for any specific intervention on attenuated positive psychotic symptoms at any time point, with the exception of ziprasidone + NBI, which was superior to NBI alone, CBT-F + NBI, and risperidone + CBT-F + NBI. However, the evidence specifically relating to ziprasidone + NBI was based on a single study only and did not survive sensitivity analyses. The results were not affected by inconsistency or evident small-study effects (publication biases).

The main finding of the current study is that there is a lack of evidence to favor specific effective interventions for reducing attenuated positive psychotic symptoms in CHR-P individuals. While ziprasidone + NBI demonstrated some superiority in the 6 month network meta-analyses, these results are not robust. First, the efficacy of ziprasidone + NBI comes from only one as-yet unpublished study. Second, the results did not survive most of the sensitivity analyses. Finally, in the cluster ranking, it was clear that while ziprasidone + NBI was the most efficacious in reducing attenuated positive symptoms, it was poorly tolerated with the lowest ranking for acceptability. Similarly, the only significant result in pairwise analyses was for CBT-F + NBI vs. NBI at 12 months. Again, this was found to be reliant on the inclusion of one particular study (74) in sensitivity analyses. Given that the data relating to the CBT-F + NBI element are identical in both the pairwise and network meta-analyses, the driving factor for the disparity (in significance of CBT-F + NBI vs. NBI in pairwise vs. network meta-analyses) likely emerges from the additional data about NBI that the NMA had gained from the rest of the network (i.e., the relative effectiveness of NBI as derived indirectly from the other -direct- comparisons). Support for this explanation comes from the finding that, when one particular study (27) was removed from the 12 month network (in sensitivity analyses), the CBT-F + NBI vs. NBI comparison became significant. Inspection of the data for this removed study (27) showed that it had the largest NBI arm (*N* = 151) of all trials, and although the study-specific SMD was not significant, the SMD was favoring NBI over the comparative intervention (omega-3 + NBI). This suggests that the relative effectiveness of NBI may have been underestimated by the direct (pairwise) CBT-F + NBI vs. NBI estimates compared to the NMA-derived estimates.

Overall, our negative results are concordant with several lines of evidence pointing toward ineffective treatments for CHR-P individuals. Beyond the lack of evidence for specific treatments reducing the risk of developing psychosis -as determined by our earlier study (22)-, another recently published network meta-analysis found no evidence that any treatments were better than any others in improving attenuated negative symptoms in CHR-P individuals (78, 79). The lack of impact on attenuated negative symptoms is in line with meta-analytical evidence showing that full-blown negative symptoms are refractory to any kind of treatment (72). More to the point, there is not even evidence that current preventive treatments can ameliorate clinical outcomes such as functional level (80–83), depressive comorbidities (83), distress (81) and quality of life (81, 83) in CHR-P individuals. It is possible that the lack of evidence for effective treatments to reduce transition to psychosis may be secondary to low statistical power for testing this outcome. In turn, this can be caused by the recruitment strategies adopted by recent RCTs that have focused on individuals that were poorly risk enriched, causing a dilution of the final risk for psychosis (23). On the contrary, the lack of evidence for effects on attenuated positive psychotic symptoms cannot simply be attributed to low statistical power. Rather, it is possible that the available treatments are not disease-modifying because they are not targeting the core pathophysiological processes underlying the onset of psychosis in CHR-P individuals (3). It is also possible that effective preventive treatments do exist, but we are currently unable to detect them because of the large noise and between-subject heterogeneity that is observed. For example, the level of attenuated positive psychotic symptoms varies considerably across different CHR-P subgroups. We have previously found that CHR-P individuals meeting the short-lived psychotic episode subgroup have the highest risk of developing psychosis (about 40–50% at 2 years) (9, 84), those meeting the attenuated psychotic symptoms subgroup have an intermediate risk (about 20% at 2 years) (9), and those meeting the genetic risk subgroup have a low risk (about 3% at 2 years) (9). In a subsequent prospective cohort study, we confirmed that CHR-P individuals meeting the short-lived psychotic episode subgroup criteria have a very high risk of developing persistent psychotic episodes (85). Additional ongoing analyses revealed that these three subgroups are associated with different clinical needs and use of mental health services. These results led us to propose clinical stratification of the CHR-P population across different subgroups (1), which has been endorsed by other leading researchers in this area (2, 86). However, because most trials were conducted before such knowledge emerged, response to preventive treatment was not stratified across these different subgroups and we have been unable to control for this variable in meta-regression analyses. The clinical heterogeneity of this population is further amplified by the heterogeneous accumulation of risk factors for psychosis (5), which is reflected in a variable enrichment of risk to psychosis (17) and different clinical outcomes. The latter may include the development of psychosis, persistence of symptoms and comorbidities, or recovery (32). Overall, the above considerations indicate that the “one-size-fits-all” approach to offering preventative strategies to CHR-P individuals is unlikely to work, namely due to the heterogeneity of the CHR-P state. This raises the possibility that the available treatments have been ineffective because they were applied to all CHR-P subjects, rather than to stratified subgroups. For example, a true preventive effect may be difficult to detect in those at low risk or in those who are responding to placebo or low-level needs-based interventions.

These findings may be informative for future research. For example, they suggest that a stratified precision medicine approach may improve the apparent effectiveness of available treatments. Identifying specific factors that predict response to preventive treatments at the individual subject level may substantially advance clinical care for CHR-P individuals by personalizing their preventive interventions. This could be achieved using the existing RCT data under an individual participant data network meta-analytic approach. These advanced meta-analytical approaches allow the stratification of treatment response through the development of predictive risk estimation tools (87) and could potentially produce a breakthrough advancement of clinical knowledge in this area. Our research group has recently completed the protocol for an individual participant data network meta-analyses (PROSPERO 2018 CRD42018089161) which is due to start imminently. At the same time, the lack of convincing evidence for effective treatments should foster refreshed collaborative efforts to test innovative novel treatments for CHR-P individuals. It is important to note that challenges in developing effective preventive treatments are not specific to the CHR-P field but are common across other branches of clinical medicine, such as in the prevention of dementia. Promising compounds are on the horizon. For example, the first ever industry-funded RCT for CHR-P individuals will be investigating the efficacy of a phosphodiesterase inhibitor to prevent psychosis (88). Of relevance, to partially reduce the clinical heterogeneity discussed above, this RCT will focus only on CHR-P individuals presenting with attenuated positive psychotic symptoms and who are enriched in risk as determined by a specific risk stratification algorithm (89). Another promising candidate treatment is cannabidiol, which was found to be well tolerated and reduced symptoms in an early-phase trial in CHR-P individuals, although the full report is not yet available (90, 91). A larger-scale RCT of cannabidiol is due to start at our institute in the near future. The discovery and development of more effective treatments for attenuated positive psychotic symptoms also requires an improved regulatory platform to reliably sustain the next generation of research. For example, while the DSM-5 includes a newly introduced diagnostic category for attenuated psychosis syndrome (92), there will be no similar diagnostic category in the ICD-11. Diagnostic controversies, as well as different methods of ascertainment of attenuated psychotic symptoms [for a comparative analysis of different CHR-P instruments see (34)] are unlikely to facilitate the large-scale collaborations that are necessary to overcome the current limitations.

There are some important limitations to our work. First, the interpretation of negative findings is always challenging. In fact, as noted by leading authors, absence of evidence is not evidence of absence (93). Such an observation is particularly relevant in the case of large CIs, such as those that have been observed in the current analyses (see Tables 3, 4). Therefore, some sizeable effects may still have been missed by our analyses. Furthermore, only 14 RCTs were included, reflecting the scarcity of studies available in this field. Although network meta-analyses are characterized by increased power and precision (94), the geometry of the networks in the current study limited our ability to test for inconsistency, and potentially resulted in more imprecise estimates and wide 95% CIs. An additional limitation is that the overall quality of our network meta-analysis is dependent on the quality of each included study, most of which were at high or unclear risk of bias. We partially controlled for this problem through assessment of biases and sensitivity analyses. The final limitation concerns the use of dropout for any reason as a proxy measure for acceptability. While this measure is generally accepted in network meta-analyses of RCTs (56–58), it is a rather crude and spurious outcome measure. The use of a more specific side effect outcome could have revealed more subtle differences in acceptability across the available treatments. We have been unable to analyse any specific side effects because these were infrequently reported in the available literature.

## Conclusions

In conclusion, on the basis of the most comprehensive evidence synthesis to date, there is currently no robust evidence to favor specific interventions for improving attenuated positive psychotic symptoms in CHR-P individuals.

## Author contributions

All authors made substantial contributions to the conception or design of the work, or the acquisition, analysis or interpretation of data. PF-P designed the study; AC optimized the study; CD and UP conducted the literature search and data extraction; JR extracted the digital data; CD and PF-P conducted the analyses under the supervision of AC and DS; CD and PF-P wrote the first draft of the manuscript; PM reviewed the draft of the manuscript.

### Conflict of interest statement

PM has received research funding from Janssen, Sunovion, GW, Boehringer Ingelheim and Roche outside of this work. PF-P has received advisory consultancy fees from Lundbeck outside of this work. The remaining authors declare that the research was conducted in the absence of any commercial or financial relationships that could be construed as a potential conflict of interest.
