# Supplementary material for: Efficacy and Acceptability of Interventions for Attenuated Positive Psychotic Symptoms in Individuals at Clinical High Risk of Psychosis: A Network Meta-Analysis
Source: Front Psychiatry. 2018 Jun 12;9:187. doi: 10.3389/fpsyt.2018.00187 (PMC6005890; doi:10.3389/fpsyt.2018.00187)
Supplement: Supplementary file 1 [file Image_1.PDF]

Supplementary Material

Efficacy and acceptability of interventions for attenuated positive psychotic symptoms in individuals at clinical high risk of psychosis: a network meta-analysis

Cathy Davies*, Joaquim Radua, Andrea Cipriani, Daniel Stahl, Umberto Provenzani, Philip McGuire, Paolo Fusar-Poli

*** Correspondence:** Cathy Davies, cathy.davies@kcl.ac.uk

Figure S1. Comparison-adjusted funnel plots for 6 (panel A) and 12 (panel B) month networks. The comparison-adjusted funnel plots present the centred effect size for pairwise comparisons of an 'active' (or newer or experimental) treatment against a 'reference' treatment (reference designated as NBI, or for those without NBI within its pairwise comparison, CBT-F+NBI), i.e. each pairwise comparison was ordered as newer/active vs NBI/CBT-F+NBI. Each observation is the difference between a study estimate and its direct meta-analytic mean effect. Different colours correspond to different comparisons. The zero (red) line represents the null hypothesis, where the study-specific effect size does not differ from the respective comparison-specific pooled effect estimate. Studies on the right hand side of the plots 'overestimate' the effect of the reference (e.g. NBI) treatment. Studies on the left hand side of the plots 'overestimate' the effect of the active (or newer or experimental) treatment. The green line represents the regression line. CBT-F – cognitive behavioural therapy (French & Morrison protocol), Dser – D-serine, Om3 – omega-3 fatty acids, NBI – needs-based interventions (including placebo), IPI – integrated psychological interventions, ARI – aripiprazole, OLA – olanzapine, RIS – risperidone, FFT – family-focused therapy, ZIP – ziprasidone.

(A)

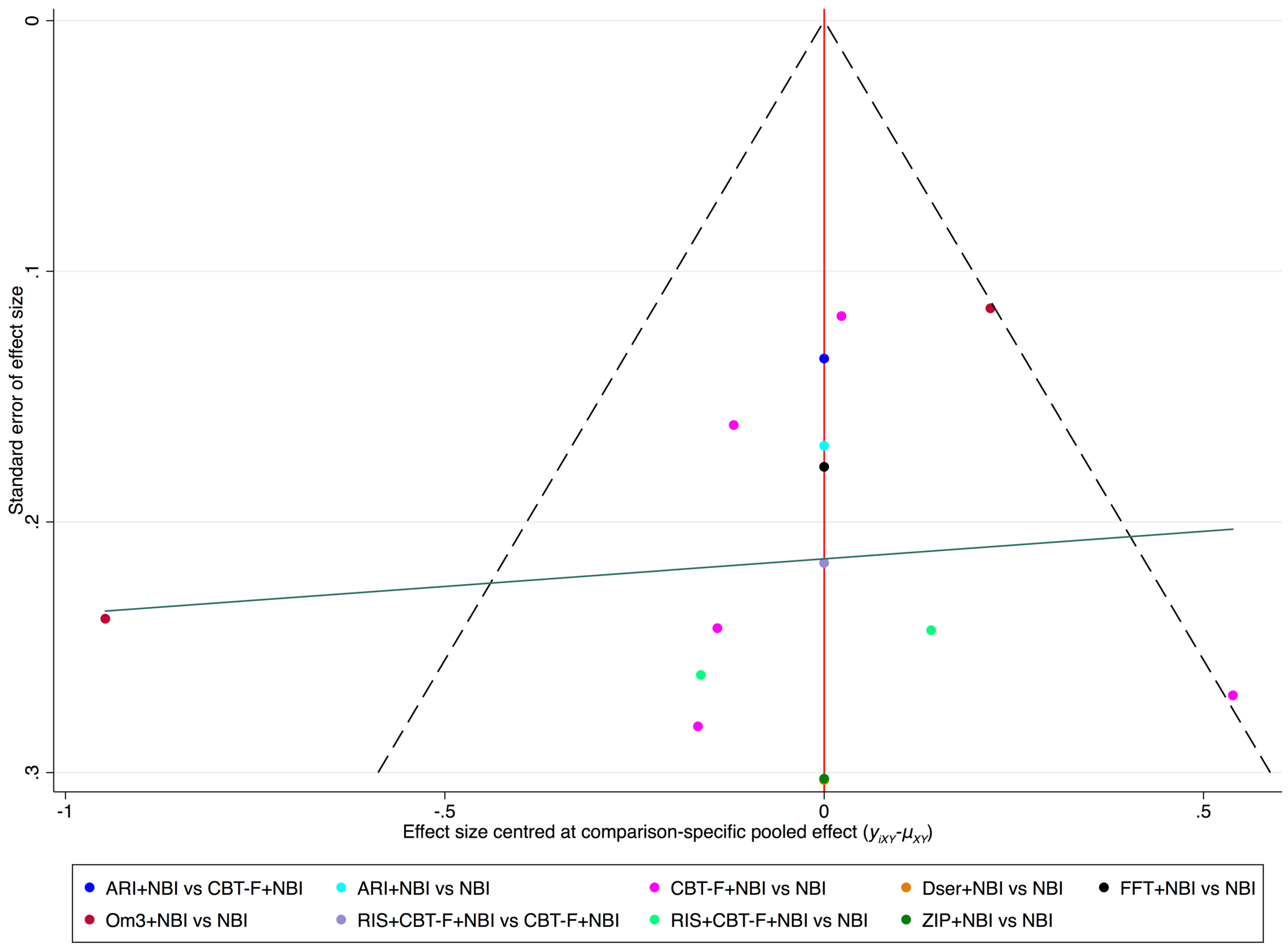

(B)

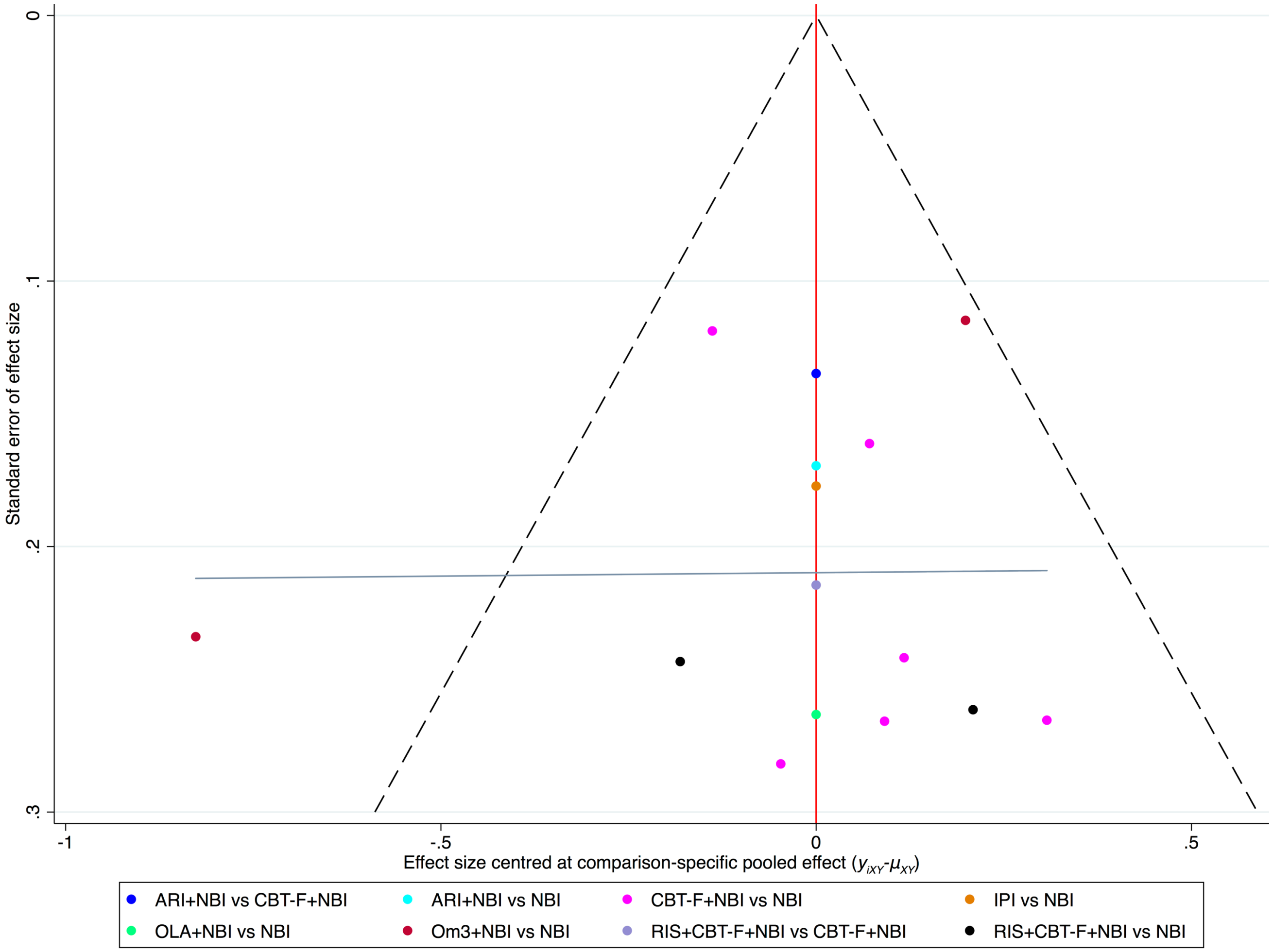

0
.1
.2
.3
Standard error of effect size
-1
-.5
0
.5
Effect size centred at comparison-specific pooled effect (y_{iXY}-μ_{XY})
ARI+NBI vs CBT-F+NBI
ARI+NBI vs NBI
CBT-F+NBI vs NBI
IPI vs NBI
OLA+NBI vs NBI
Om3+NBI vs NBI
RIS+CBT-F+NBI vs CBT-F+NBI
RIS+CBT-F+NBI vs NBI
